# Transfemoral transcatheter aortic valve implantation: towards local anaesthesia for everyone?

**DOI:** 10.1007/s12471-022-01728-1

**Published:** 2022-10-24

**Authors:** A. C. van Nieuwkerk, J. J. Piek

**Affiliations:** Department of Cardiology, Amsterdam Cardiovascular Sciences, Amsterdam UMC, University of Amsterdam, Amsterdam, The Netherlands

Nationwide registries play a central role in cardiovascular research. In addition to performance and quality management, registry data provide real-world insights into clinical outcomes of large patient cohorts. These registries represent the full spectrum of patients, including frail patients who are underrepresented in clinical trials. The Netherlands Heart Registration (NHR) was initiated in 2017 for quality-of-care purposes and reporting is mandatory for all centres that perform transcatheter aortic valve implantation (TAVI) in the Netherlands.

In this issue of the *Netherlands Heart Journal*, Rooijakkers et al. describe the use of NHR data to assess the effects of the coronavirus disease 2019 (COVID-19) pandemic on clinical outcomes in TAVI patients. The authors compared the outcomes of patients treated during the first COVID-19 wave (March to July 2020) with those of patients treated a year before the COVID-19 pandemic (March to July 2019). Rates of clinical outcomes and mortality were similar up to 150 days after TAVI in these two patient cohorts [1]. Their results show that TAVI can be safely performed during the COVID-19 pandemic. These data are especially reassuring in light of potential future pandemics or other situations where resources are scarce. Interestingly, Rooijakkers et al. also observed a trend towards increased use of local anaesthesia rather than general anaesthesia in the COVID-19 cohort. Rates of general anaesthesia decreased from 46.5 to 35.2% (*p* < 0.001). Assuming all 301 non-transfemoral TAVI patients (transaxillary, transapical and direct transaortic) in the study population underwent general anaesthesia, 32.9% (pre-COVID-19) and 20.4% (COVID-19) of transfemoral TAVI procedures were still performed using general anaesthesia. It would be of additional interest to assess the clinical outcomes in patients treated with local versus general anaesthesia.

The current 2021 European Society of Cardiology and the 2020 American College of Cardiology/American Heart Association guidelines do not give recommendations about anaesthesia type in transfemoral TAVI. The randomised SOLVE-TAVI trial showed that local anaesthesia with conscious sedation was non-inferior to general anaesthesia for a composite endpoint of 30-day mortality, stroke, myocardial infarction, infection and acute kidney injury [2]. However, the study was underpowered for individual endpoints. Observational data from the United States Transcatheter Valve Therapy Registry showed that the use of conscious sedation varies between hospitals from 0 to 91%. The use of conscious sedation in this U.S. registry increased from 33% in 2016 to 64% in 2019 and was associated with a 0.2–0.5% lower risk of short-term mortality [3]. Conscious sedation includes local anaesthesia with light to moderate sedation, but without intubation [2, 3]. Unfortunately the Dutch NHR data do not distinguish between local anaesthesia with versus without conscious sedation.

Local anaesthesia may result in quicker recovery and a shorter hospital stay [2, 3]. A shorter hospitalisation reduces the strain on hospital resources, which is particularly important during a pandemic. Moreover, periprocedural stroke can be detected earlier during local anaesthesia, and may be treated accordingly. The general TAVI patient population is frequently old and frail. There is a delicate haemodynamic balance in these patients which may be disturbed during the TAVI procedure by rapid pacing, obstruction of the stenotic valve by the device before implantation, and during valve placement. Moreover, induction of general anaesthesia itself may disturb haemodynamic stability. Recovery in patients with impaired baseline haemodynamic stability can take longer, e.g. in patients with impaired left ventricular ejection fraction and/or susceptibility to acute heart failure. Although available evidence assumes local anaesthesia to be safe, one could argue that general anaesthesia may be safer in some patients. In local anaesthesia, patients need to be clearly instructed to remain stable in a supine position, which can be challenging in cognitively impaired, severely stressed or orthopnoeic patients.

Similar to the early years of percutaneous coronary intervention, there is an ongoing trend to minimise the complexity of TAVI procedures: percutaneous vascular access instead of surgical cut-down, and same- or next-day discharge [4]. Currently there are no data from the Netherlands on whether this trend towards the use of local anaesthesia and conscious sedation is continuing after 2020 and whether the COVID-19 pandemic may have accelerated this trend. The type of anaesthesia used in TAVI depends on local practice and preferences. However, results from our own Amsterdam UMC database, where local anaesthesia is the default strategy for performing TAVI, are reassuring [5]. Local anaesthesia results in leaner and less invasive TAVI procedures with a shorter recovery time [4]. Patients are able to start mobilising after a few hours, which potentially has many advantages in this frail and elderly population. Early mobilisation can lower rates of delirium, infection, reported pain and unplanned urinary catheter use [5]. Moreover, local anaesthesia can facilitate the implementation of early discharge programmes, reducing the strain on hospital resources even further [4].

In conclusion, NHR data confirm that TAVI can be safely performed during the COVID-19 pandemic in the Netherlands. Moreover, the observed trend towards increased use of local anaesthesia in TAVI procedures is a promising new development that may reduce the strain on hospital resources, relevant in the anticipation of a new pandemic, while improving clinical outcomes in patients undergoing TAVI.
